# The One Health Approach to Toxoplasmosis: Epidemiology, Control, and Prevention Strategies

**DOI:** 10.1007/s10393-019-01405-7

**Published:** 2019-04-03

**Authors:** A. Alonso Aguirre, Travis Longcore, Michelle Barbieri, Haydee Dabritz, Dolores Hill, Patrice N. Klein, Christopher Lepczyk, Emily L. Lilly, Rima McLeod, Judith Milcarsky, Caroline E. Murphy, Chunlei Su, Elizabeth VanWormer, Robert Yolken, Grant C. Sizemore

**Affiliations:** 1 Department of Environmental Science and Policy, George Mason University, 4400 University Dr. MSN: 5F2, Fairfax, VA 22030-4400 USA; 2 Spatial Sciences Institute, University of Southern California, 3616 Trousdale Parkway, AHF B55, Los Angeles, CA 90089 USA; 3 NMFS/PIFSC/PSD/Hawaiian Monk Seal Research Program, 1845 Wasp Boulevard, Building 176, Honolulu, HI 96818 USA; 4 Community Health Branch, Yolo County Health & Human Services Agency, 137 N Cottonwood St, Woodland, CA 95695 USA; 5 U.S. Department of Agriculture, Center Road Building 307-C Room 134, BARC East, Beltsville, MD 20705 USA; 6 United States Department of Agriculture Forest Service, 201 14th Street, SW, Washington, DC 20250 USA; 7 Auburn University, SWFS 2341, 602 Duncan Drive, Auburn, AL 36849 USA; 8 Virginia Military Institute, 303D Maury-Brooke Hall, Lexington, VA 24450 USA; 9 The University of Chicago, AMB N310, (MC 2114) 5841 South Maryland Avenue, Chicago, IL 60637 USA; 10 The House-Call Vet, Daytona Beach, FL 32114 USA; 11 The Wildlife Society, 425 Barlow Place, Suite 200, Bethesda, MD 20814 USA; 12 M409 Walters Life Sciences, University of Tennessee, Knoxville, TN 37996 USA; 13 University of Nebraska-Lincoln, 406 Hardin Hall, 3310 Holdrege Street, Lincoln, NE 68583 USA; 14 Stanley Neurovirology Laboratory, Johns Hopkins School of Medicine, Baltimore, MD 21287 USA; 15 American Bird Conservancy, 4301 Connecticut Ave., NW, Suite 451, Washington, DC 20008 USA

**Keywords:** One Health, *Toxoplasma gondii*, Toxoplasmosis, Transdisciplinarity, Integrative research

## Abstract

One Health is a collaborative, interdisciplinary effort that seeks optimal health for people, animals, plants, and the environment. Toxoplasmosis, caused by *Toxoplasma gondii*, is an intracellular protozoan infection distributed worldwide, with a heteroxenous life cycle that practically affects all homeotherms and in which felines act as definitive reservoirs. Herein, we review the natural history of *T. gondii*, its transmission and impacts in humans, domestic animals, wildlife both terrestrial and aquatic, and ecosystems. The epidemiology, prevention, and control strategies are reviewed, with the objective of facilitating awareness of this disease and promoting transdisciplinary collaborations, integrative research, and capacity building among universities, government agencies, NGOs, policy makers, practicing physicians, veterinarians, and the general public.

## Introduction

Toxoplasmosis, caused by infection with the coccidian *Toxoplasma gondii*, is a significant public health problem worldwide. An estimated 8–22% of people in the USA are infected, and similar prevalence exists in the UK (Dubey 2002; Dubey and Jones 2008; Jones et al. 2001, 2003, 2007). In Central America, South America, and continental Europe, estimates of infection range from 30 to 90% (Dubey and Jones 2008; Dubey 2010; Minbaeva et al. 2013; Wilking et al. 2016).

These infections have significant consequences affecting mortality and quality of life. In the USA, where over a million people are infected each year and approximately 2839 people develop symptomatic ocular disease annually, the cost of illness has been estimated to be nearly $3 billion and an 11,000 quality-adjusted life-year loss annually (Jones and Holland 2010; Batz et al. 2012; Hoffmann et al. 2012). Mead et al. (1999) suggested that *T. gondii* is one of three pathogens (along with *Salmonella* and *Listeria*) that account for > 75% of all deaths due to foodborne disease in the USA. Scallan et al. (2011) estimated that *Toxoplasma* caused 8% of hospitalizations and 24% of deaths in the USA resulting from foodborne illnesses.

As a global strategy, One Health recognizes the interconnectedness of the health of people, animals, plants, and the environment from the local to the global levels and employs a holistic approach encouraging and expanding transdisciplinary collaborations, integrative research, capacity building, clinical practice, policy, and communication among many stakeholders. This approach can overcome bureaucratic boundaries and represents an opportunity for new partnerships focused on solutions for humans, animals, plants, and the environment (Zinsstag 2012; Rubin et al. 2014; Aguirre et al. 2016). Toxoplasmosis qualifies as a One Health disease because it significantly affects the health of human, domestic animals, wildlife, and ecosystems, and is perceived as a threat by those who rely on animal resources (Crozier and Schulte-Hostedde 2014; Jenkins et al. 2015). The complicated relationships across taxa are compounded by changing practices and attitudes toward the control of owned and unowned (stray and feral) outdoor domestic cats (*Felis catus*), which are the obligate reservoirs of the parasite in urban and suburban settings, where native wild felids are largely absent (Afonso et al. 2008).

New research on the impacts of toxoplasmosis (Ngo et al. 2017; Suvisaari et al. 2017) increases the need for greater institutional awareness of the pathways of infection and comprehensive and transdisciplinary actions to control transmission using the One Health approach. Such cooperation has thus far been elusive, perhaps in part to a lack of familiarity with the biology of *T. gondii* or its significant adverse impacts on health (Efunshile et al. 2017). Herein, we review the natural history of *T. gondii*, its transmission and impacts, and suggest approaches that could help protect human, domestic animal, wildlife, and ecosystem health, with the goal of facilitating a better understanding of this disease and promoting transdisciplinary collaborations, integrative research, and capacity building among universities, government agencies, NGOs, policy makers, physicians, veterinarians, and the general public.

## Natural History of *Toxoplasma gondii*

*Toxoplasma gondii* is a member of the Apicomplexa, a diverse group of parasitic protozoans including *Babesia, Cryptosporidium*, *Cyclospora*, *Isospora*, and *Plasmodium* (Kim and Weiss 2004). It was first isolated from a common gundi (*Ctenodactylus gundi*) in Tunis in 1908 and the same year in a rabbit from South America. Six clades have been characterized using population genetic structure studies indicating that globally diverse isolates originate from a small number of ancestral lineages (Su et al. 2012). It is postulated that *T. gondii* originated in South American felids with relatively recent expansion through migratory birds and in particular the transatlantic slave trade that promoted migration of domestic cats, rats, and mice (Lehmann et al. 2006). Three predominant archetypal clonal lineages of *T. gondii* have been identified (Howe and Sibley 1995; Ajzenberg et al. 2004; Dardé 2004; Saeij et al. 2005). Diverse atypical genotypes have also been found in the Americas and China (Miller et al. 2008; Khan et al. 2011; Chaichan et al. 2017). Shwab et al. (2014) used 10 PCR–RFLP markers to classify 1457 *T. gondii* specimens into 189 genotypes, most of which fell into genotypes 1 through 5. Although no dominant genotype has been found in the southern hemisphere, a few genotypes were predominant in the northern hemisphere, specifically genotypes 1 (type II clonal), 2 (type III), and 3 (type II variant), which comprise the majority of isolates and are prevalent in Europe. Genotypes 2 to 5 (4 and 5 collectively known as type 12, and prevalent in wildlife) are common in North America. Genotypes 2 and 3 predominated in Africa, whereas genotypes 9 and 10 were highly prevalent in China (Wendte et al. 2011; Shwab et al. 2014; Chaichan et al. 2017). Certain genotypes are associated with increased virulence in humans and wildlife (Sibley and Boothroyd 1992; Miller et al. 2004; Carme et al. 2009; Xiao and Yolken 2015). Lorenzi et al. (2016) compared the genomes of 62 globally distributed isolates, identifying that *T. gondii* is characterized by clade-specific inheritance of large conserved haploblocks with different ancestries that may influence transmission, host range, and pathogenicity. Clonal lineages 1–4 are extremely abundant, with highly similar multilocus genotypes, high levels of linkage disequilibrium, and infrequent recombination.

**Figure 1:**
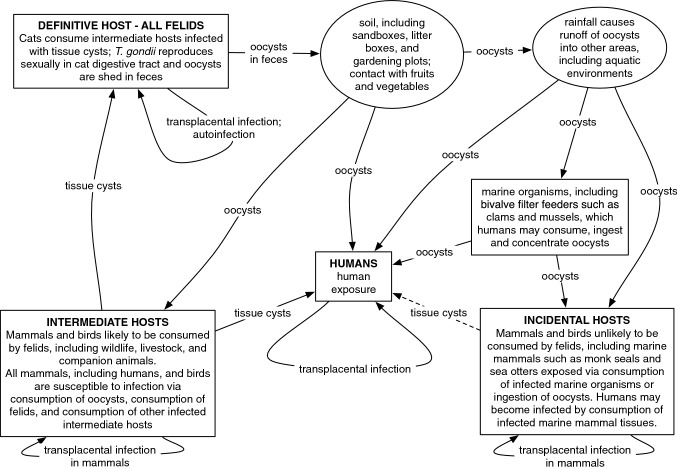
Life cycle of *Toxoplasma gondii* and transmission in humans, domestic animals, wildlife and ecosystems

The parasite can only sexually reproduce and, thereby, complete its life cycle in felids, which are definitive hosts capable of excreting massive numbers of oocysts into the environment via feces (Frenkel 1973). Oocysts will sporulate to contain infectious sporozoites. If as few as a single sporulated oocyst is ingested or inhaled by an intermediate host, including all classes of warm-blooded (homeotherm) vertebrates, *T. gondii* may then reproduce asexually in the host’s tissues (Miller et al. 1972; Dubey et al. 1996). Asexual reproduction results in the formation of tachyzoites and bradyzoites from sporozoites. While bradyzoites form tissue cysts in the intermediate host, tachyzoites invade many host tissues to include the heart, lung, and central nervous system and will spread by intrauterine infection and transplacental migration to infect the fetus; (Georgi 1985; Markell 1986). The life cycle is completed when the tissues of an intermediate host are consumed by a cat, and sexual reproduction in the definitive host may begin again (Fig. 1). One of the ways by which *T. gondii* facilitates the completion of its life cycle is host manipulation. Infected rodents, for example, lose their innate fear of cats and demonstrate an attraction to cat urine (Berdoy et al. 2000; Vyas et al. 2007). Host manipulations associated with *T. gondii* infection have also been observed or hypothesized in other taxa, including primates and birds (Poirotte et al. 2016; Work et al. 2016).

### Toxoplasmosis in Humans

Toxoplasmosis is the second leading cause of death among foodborne illnesses in the USA (Scallan et al. 2011; Gao et al. 2016). In humans, symptoms, or lack thereof, at the time of infection do not predict disease manifestation later in life. The disease may be either acute or chronic and can cause active infection at any age (Boyer et al. 2011; Delair et al. 2011). Postnatal *T. gondii* infection may appear to be asymptomatic or cause fever and lymphadenopathy (Montoya and Remington 1995) and affect any organ, especially the eyes (Delair et al. 2011; Undseth et al. 2014), and cause seizures (McAuley et al. 1994). Virulence varies by strain and susceptibility based on an individual’s genetic traits (Ngo et al. 2017). Genotypes in French Guiana, for example, cause significant damage and even death in adults who are not known to be immunocompromised (Carme et al. 2009). In the USA, an estimated 1.1 million people are infected with *T. gondii* each year, and approximately 10.4% of the population demonstrate seroprevalence linked to past exposure (Jones and Holland 2010; Jones et al. 2018).

Initial infection acquired by pregnant women may cross the placenta and reach the fetus (McLeod et al. 2014). Toxoplasma tachyzoites multiply and invade fetal tissues to cause acute or chronic disease (Markell 1986). This congenital infection may be systemic and result in fetal death, premature birth, intrauterine growth retardation, fever, pneumonia, hepatosplenomegaly, thrombocytopenia, or involve the eyes and brain (McAuley et al. 1994; Peyron et al. 2016). Manifestations of ocular or encephalic disease in the fetus may include chorioretinitis, meningoencephalitis, hydrocephaly, microcephaly, or calcifications of previous areas of necrosis; however, infants generally do not show clinical signs at birth and instead may become deaf later in life. When women acquire the infection more than 6 months prior to gestation, risk of transmission to the fetus is considerably reduced. Although preventable and treatable, congenital, ocular, and postnatal *T. gondii* infection is not curable and persists in all infected persons (Ortiz et al. 2013; Peyron et al. 2016; Ngo et al. 2017). Latent or primary toxoplasmosis can be particularly dangerous in individuals with compromised immune systems, including those treated with corticosteroids, cytotoxic medicines, and antibody to tumor necrosis factor alpha (Lykins et al. 2016; Wang et al. 2017). Approximately one third of HIV-infected individuals with *T. gondii* infection develop encephalitis (Walker and Zunt 2005).

Retinal toxoplasmosis (Kianersi et al. 2012) is recognized as a major cause of blindness in many parts of the world (Balasundaram et al. 2010). About 5000 people develop ocular toxoplasmosis in the USA annually (Jones and Holland 2010). Chronic infections, previously believed to be benign, are now a source of increasing concern. Evidence of exposure to *T. gondii* has been associated with cognitive decline in older individuals (Gajewski et al. 2014) and increased disease overall (Flegr et al. 2014). Furthermore, such serological evidence of *T. gondii* is associated with a range of neuropsychiatric disorders including schizophrenia (Torrey and Yolken 2003; Yolken et al. 2009), depression, suicide attempts (Arling et al. 2009; Flegr et al. 2014), and anxiety disorders. The mechanisms that define these associations are not known with certainty but may be related to the immune response to the tissue cysts and presence of bradyzoite tissue cysts within the brain following infection (Xiao et al. 2016).

Recent studies have demonstrated that undetected environmental oocyst transmission is the major route of *T. gondii* transmission presenting a direct public and animal health problem (Tenter et al. 2000, Dabritz and Conrad 2010, Boyer et al. 2011, Hill et al. 2005, 2011, Torrey and Yolken 2013; VanWormer et al. 2016). The risk factors for human and animal infection include consuming infected raw or undercooked meat; ingestion of contaminated water, soil, vegetables, or anything contaminated with oocysts shed in feces; blood transfusion or organ transplants; intrauterine or transplacental transmission; and drinking infected unpasteurized milk. The majority (78%) of congenital toxoplasmosis cases from four epidemics in North America originated from oocyst exposure, though only 49% of these cases could be confirmed as foodborne. Two public health studies in Chile evaluated oocyst-acquired infections in pregnant women and in swine, which are a primary food source; *T. gondii* oocyst-specific IgG antibodies were determined in 193/490 (43%) of serum samples from pregnant women and in 24/30 (80%) of 30/340 (8.8%) the swine (Muñoz-Zanzi et al. 2010, 2012). Oocysts can also contaminate drinking water sources, both small-scale wells (Sroka et al. 2006) and larger reservoirs (Bowie et al. 1997), and can contaminate surfaces, such as dog fur (Frenkel et al. 2003) or keypads (Bik et al. 2016).

### Toxoplasmosis in Domestic Animals

Despite a high global prevalence, infected domestic cats typically are asymptomatic and do not have recognizable clinical disease (Hill and Dubey 2014). Nevertheless, clinical signs may include fever, ocular inflammation, anorexia, lethargy, pneumonia, abdominal discomfort, and central nervous system disturbances (Vollaire et al. 2005; Dubey and Jones 2008). Clinical infection is most severe in kittens, and feral domestic cats are at a higher risk of infection than indoor cats (Dubey and Jones 2008).

Domestic dogs may also be infected with *T. gondii*; however, clinical infection is less common than subclinical disease (Dubey et al. 2009; Hill and Dubey 2013). When manifested, clinical signs may affect respiratory, neuromuscular, or gastrointestinal systems and can prove fatal (Dubey et al. 2009). Free-roaming dogs are believed to be at higher risk, though dogs may become infected within households by eating uncooked infected meat (Cabezón et al. 2010).

Toxoplasmosis is common in sheep, goats, pigs, and chickens as intermediate hosts; however, cattle and horses are notably resistant to the disease. In sheep, congenital infection is a leading cause of stillbirth and preterm lamb loss. Lambs that are born infected and survive usually exhibit normal growth, but they still represent a public health risk if their infected meat is consumed (Dubey 2009). Toxoplasmosis can also occur in adult goats, and the disease is more severe than in sheep. Congenital infection results in loss of kids before or after birth. Pigs may become infected with *T. gondii* by consumption of oocysts, congenitally by tachyzoite transplacental transmission, and through consumption of meat containing *T. gondii* bradyzoite tissue cysts. Although adult pigs rarely show clinical signs, the meat of infected pigs serves as a source of human infection; young pigs can die from toxoplasmosis without entering the human food chain. Animal infections with *T. gondii* appear to be largely driven by environmental exposure to the oocysts, and the presence of outdoor domestic cats has been identified as risk factor for infection in farm animals (Vesco et al. 2007). Consequently, non-confinement livestock housing and facilities lacking adequate biosecurity and pest management practices represent significant risk factors for livestock infection (Dubey and Jones 2008; Hill and Dubey 2016).

### Toxoplasmosis in Wildlife

Toxoplasmosis is a global disease found in all habitats and regions, from the Arctic to the tropics in terrestrial, aquatic, and marine settings affecting all homeotherms (Sibley 2003). The number of documented infected species is extensive (e.g., Dubey and Jones 2008; Dubey 2010). Detection in apparently healthy, free-ranging wildlife suggests that asymptomatic or subclinical infections may occur. Pathways for wildlife infection include consumption of infected felids, predation or scavenging of infected intermediate hosts, direct ingestion of oocysts in the contaminated environment, and congenital transmission by transplacental transmission of tachyzoites from the infected parent (Fig. 1). Environmental transmission to carnivores and omnivores such as polar (*Ursus maritimus*), grizzly (*Ursus arctos*), and black bears (*Ursus americanus*) can be driven by either consumption of infected meat in prey species or direct ingestion of oocysts (Chomel et al. 1995; Oksanen et al. 2009).

Depending on their geographic range, serologic studies in herbivores correlate with density of domestic cats linked to oocyst density (Fredebaugh et al. 2011). For example, Hawaiian geese (*Branta sandvicensis*) have seroprevalences of 21–48% as a result of exposure to oocysts (Work et al. 2016). Other terrestrial, wild herbivores infected by *T. gondii* include white-tailed deer (*Odocoileus virginianus*), with reported seroprevalence of 49.5% in suburban areas and 66.1% in urban areas, indicative of a greater prevalence of oocysts in the soil as domestic cat densities increase with human populations (Lélu et al. 2010; Dubey et al. 2014; Ballash et al. 2015).

Infection in marine mammals is geographically and taxonomically widespread, driven by land-to-sea coastal oocyst pollution linked to oocysts from storm water runoff (Cole et al. 2000; Dubey et al. 2003; Littnan et al. 2006; Aguirre et al. 2007; Lindsay and Dubey et al. 2009; Oksanen et al. 2009; Jensen et al. 2012; Rengifo-Herrera et al. 2012). The threatened southern sea otter (*Enhydra lutris nereis*), exposed through the consumption of invertebrate prey, (Johnson et al. 2009; Shapiro et al. 2014), serves as a sentinel of the land-to-sea flow of *T. gondii* oocysts originating from runoff carrying infected domestic or wild felid fecal matter (Jessup et al. 2004; Conrad et al. 2005). This route of exposure has been confirmed for other marine mammals (Miller et al. 2002, 2008; Conrad et al. 2005). Similar genotypes have been detected in tissues from sea otters, terrestrial wildlife, i.e., bobcats, mountain lion, and wild canids, and feral domestic cats sharing the California coast (Miller et al. 2008; VanWormer et al. 2014; Verma et al. 2017).

Aquatic invertebrates may significantly influence waterborne transport of *Toxoplasma*, by enhanced settling and subsequent benthos concentration, and by facilitating ingestion by invertebrate vectors that can transmit the infective stage to susceptible hosts, including marine mammals and humans. Recent studies by Shapiro et al. (2014) have demonstrated the critical role of invisible polymers in transmission of *T. gondii* in food webs through particle aggregates and biofilms increasing the retention of the parasite in snails grazing on kelp and facilitating infection of California sea otters.

Land-to-sea coastal exposure has resulted in fatal toxoplasmosis in phocids, otariids, mustelids, and cetaceans, negatively impacting some threatened and endangered populations (Holshuh et al. 1985; Inskeep et al. 1990; Migaki et al. 1990; Jardine and Dubey 2002; Dubey et al. 2004; Carlson-Bremer et al. 2015; Barbieri et al. 2016). Yet data on mortality in marine mammals are limited to those obtained through necropsies of stranded animals. More animals die than are found dead every year, particularly for offshore and migratory taxa; hence, the number of affected marine mammals is likely underrepresented.

### Toxoplasmosis in Ecosystems

Domestic cats are likely the major source of ecosystem contamination in many areas due to their high abundance on the landscape relative to native felids (VanWormer et al. 2013). A large percentage of domestic cats in the USA may carry *T. gondii* during their lifetime (Tenter et al. 2000), and each infected cat sheds up to hundreds of millions of oocysts (Dubey 1996), with the high probability that any location with free-roaming cats will become contaminated with oocysts (Torrey and Yolken 2013). Each oocyst may remain infectious for months to years (Tenter et al. 2000; Lélu et al. 2012). Dabritz et al. (2007) estimated that owned, domestic cats in Morro Bay, California, annually deposited 77.6 tons of feces and that free-roaming cats in the same area deposited 30 tons of feces, resulting in an estimated annual oocyst loading of over 4500 oocysts/m^2^. The greater the number of cats, the greater the accumulation of oocysts, and, presumably, the greater the probability of transmission to humans, other domestic animals, and wildlife.

An estimated 30–80 million feral domestic cats exist in the USA (Loss et al. 2013); all of which defecate outdoors, and each of which are at a much higher risk of hosting and spreading *T. gondii* (Dubey 2010; VanWormer et al. 2013). Unlike with other domestic animals (e.g., domestic dogs), many states do not address who or what entity is responsible for unowned and feral domestic cats. A patchwork of local or nonexistent regulations may increase confusion, hampering the ability to control domestic cats even on one’s own property. This regulatory confusion, combined with the efforts by some private organizations to eliminate all euthanasia for animal control (Longcore et al. 2009; ASPCA 2017), has contributed to the establishment of programs and policies that preserve unowned and feral domestic cats on the landscape. Such programs remain in place despite calls for the removal of unowned and feral domestic cats from the environment for a variety of reasons (e.g., public health, wildlife conservation, animal welfare) from numerous professional organizations and government agencies and evidence of public support (Levy and Crawford 2004; Lohr and Lepczyk 2014). Public opposition to removal policies may be partially influenced by misinformation that minimizes the risks of toxoplasmosis and downplays the role of domestic cats as vectors for disease transmission in many ecosystems as documented in the scientific literature (Loss and Marra 2018).

Animal sheltering policies can influence the risk of *T. gondii* transmission by affecting the number of free-roaming domestic cats. Management policies that remove cats from ecosystems reduce environmental transmission risks by eliminating the interaction of definitive and intermediate hosts. Conversely, policies that intentionally maintain unowned and feral domestic cats on the landscape facilitate and may increase the risk of disease transmission. As a growing number of municipalities and their animal shelters adopt policies that purposely maintain domestic cats unconfined outdoors (Holtz 2014), *T. gondii* transmission risks for people, domestic animals, and wildlife increase.

A better understanding of the environmental abundance of oocysts is critical to holistic determinations of health risks. Studies indicate that large regions of terrestrial, aquatic, and marine environments may be contaminated (Du et al. 2012a, b; Gao et al. 2016; VanWormer et al. 2013). *T. gondii* is known to be influenced by environmental conditions, and survival of oocysts in the soil may be influenced by geological and environmental characteristics such as soil temperature, texture, and chemistry (Frenkel et al. 1975; Lindsay and Dubey 2009; Lélu et al. 2012). Broader environmental sampling of oocysts in soil and stormwater runoff should be undertaken and modeled by land use, feline density, and animal shelter policies. The environmental parameters responsible for long-term survival and resistance of oocysts, regional extent of environmental contamination with oocysts, and duration of survival or infectivity of tissue cysts following host death are poorly understood and require additional research. The ubiquity of *T. gondii* oocysts in the environment increases the likelihood of infection for all at-risk species in the ecosystem. Perhaps the most important ecosystem management tool is to control contaminated runoff to mitigate the health impacts of coastal habitat pathogen pollution.

## Envisioning a One Health Response

One Health focuses on transdisciplinary collaborations to solve issues across human health, animal and plant health, and the environment. Accordingly, for the near- and long-term future, One Health has little choice but to engage in the study, mitigation, and prevention of daunting challenges (Aguirre et al. 2016). Critical to One Health will be effective monitoring of toxoplasmosis and *T. gondii* prevalence. In the USA, toxoplasmosis is not a nationally reportable disease and, thus, its true magnitude is unknown (Jones et al. 2001; Torgerson and Mastroiacovo 2013). Likewise, although the burden of toxoplasmosis in other countries like Brazil is very high, we can only hypothesize, for example, the incidence of congenital disease in children (Dubey et al. 2012). Although some screening of pregnant women and newborns exists in the USA, these programs are largely absent and fall behind the regular screening in many countries (Peyron et al. 2017). Furthermore, screening programs associated solely with congenital toxoplasmosis may miss large segments of the infected population. Enhanced screening programs would deliver greater data that can be used to develop more responsive tools for risk reduction. Integrating human, domestic animal, and wildlife data could better assess risk and devise methods of control.

Current patterns of human-driven environmental change and globalization of travel and trade can enhance the spillover and spillback of *Toxoplasma* and parasites of animal origin into human populations, introduce pathogens into critically endangered animal populations, and further facilitate propagation locally, regionally, and globally. The odds of an infectious disease pandemic have never been higher. Furthermore, given that most emerging infectious diseases in humans are of animal origin (zoonotic), there is a pressing need to integrate human–animal–ecosystem health within a common framework. The recent convergence of global problems, including global environmental change, biodiversity loss, habitat fragmentation, globalization, and infectious disease emergence, demands integrative approaches breaching disciplinary boundaries leading to “One Health.” This integration requires commitment not only from government agencies, universities, and other organizations but eventually will attempt to generate new international structures (Aguirre 2011; Gortazar et al. 2014; Suzán et al. 2015).

### Transdisciplinarity

Simple solutions are rarely evident in addressing regional or global ecological and environmental problems. A multi-pronged, transdisciplinary, One Health approach is required in infectious disease ecology. For example, this approach has been used in echinococcosis in North America (Massolo and Liccioli 2016); during evaluation of rabies control programs in Sri Lanka (Häsler et al. 2014); during parasitic zoonosis surveillance in Australian wildlife (Thompson 2013); and in foodborne diseases resulting from *Cryptosporidium* spp., *Giardia duodenalis*, *Cyclospora cayetanensis*, and *T. gondii*, in developed countries (Dixon 2016); in the past 10 years, new tools and institutional initiatives for assessing and monitoring emerging pathogens have been developed. Landscape epidemiology, disease ecological modeling, and web-based Google analytics have emerged. New types of integrated ecological health assessment are being deployed; these efforts incorporate environmental indicator studies with specific biomedical diagnostic tools. Other innovations include the development of noninvasive physiological and behavioral monitoring techniques, the adaptation of modern molecular biological and biomedical techniques, the design of population-level disease monitoring strategies, the creation of ecosystem-based health and sentinel species surveillance approaches, and the adaptation of health monitoring systems for appropriate low-income country situations. Ultimately, a data-driven decision support tool must be created to help practitioners and managers devise choices for action and intervention. Epidemiologists, modelers, public health officials, veterinarians, and sociobiologists need to employ strong inference techniques including model selection, disease inference techniques, to apply a rigorous approach to establishing causation in disease ecology (Azeez and Prabhakar 2016). Mathematical modeling, predictive tools, and novel prevention strategies of emerging infectious diseases have evolved enormously in the last decade. These exciting tools now allow for improved characterization and prediction of disease dynamics and disease behavior (Vinetz et al. 2005; Aguirre et al. 2016; Guo et al. 2016).

*Toxoplasma gondii* is known to be influenced by environmental conditions, and measures to mitigate exposure can affect ecosystem health. The environmental parameters responsible for long-term survival and resistance of the parasite in oocyst form or the duration of survival or infectivity of tissue cysts from an infected animal that dies in the field are poorly understood. Filling these science and knowledge gaps will require effective, truly transdisciplinary collaborations involving scientists from a broad spectrum of disciplines including but not limited to earth, environmental, biological, ecological, social, engineering, and health sciences, and their many subdisciplines (Aguirre and Wilcox 2008).

### Integrative Research

Research is needed to integrate data across scales to assess risk and devise methods of control, as links are made between toxoplasmosis and significant adverse health outcomes beyond acute infection in humans, i.e., congenital infection, increased death rates in traffic accidents (Flegr et al. 2002, 2009), and environmental transmission rather than meat consumption emerges as a significant pathway for infection (Dabritz and Conrad 2010; Boyer et al. 2011; Hill et al. 2011; Torrey and Yolken 2013). Soil sampling for oocysts has been undertaken around the world and needs to be expanded and modeled by land use and outdoor cat management policies to understand risk. Relationships of policies for animal sheltering and outdoor cat density and resulting oocyst loads are foreseeable but require integrative research. Such research efforts will require transdisciplinary teams to integrate field and laboratory methods, spatial, geographic, and other mathematical modeling, and veterinary and medical practices.

Future research should also focus on vaccine development. A vaccine is available for sheep in some countries, but no vaccine exists for other livestock, humans, or wildlife. A vaccine for domestic cats was produced, but its implementation has been limited by high costs of production, short shelf life, and lack of interest from domestic cat owners (Dubey 2010). The development of a vaccine, as well as more effective therapies for the long-term effects of tissue cysts in the brain, eye, and other vital organs, remain important goals. Such research would also benefit efforts to conserve highly endangered species in the wild that are at risk from death from toxoplasmosis (Work et al. 2016).

The increasing demand for food safety together with the potential economic impact of legislation aimed at risk reduction has brought attention to the need for the development and standardization of diagnostic tests for *Toxoplasma* infection. Such tests will need to provide an accurate estimate of risks of transmission of toxoplasmosis to humans and must perform with comparable specificity and sensitivity across a range of animal species. Despite the lack of widespread, effective screening processes are in place for consumer meats, with new standardized tests which may be useful for disease monitoring and control (Nunes Mecca et al. 2011).

### Building Local Capacity

A key component of a One Health response to toxoplasmosis must include greater communication of the risks and pathways of exposure to *T. gondii*. Human and veterinary health practitioners, as well as all professionals interacting with the public, should seek to more effectively explain current understandings of the life history, transmission routes, and best practices for avoiding exposure. For example, while acknowledging the risks of infection through consumption of tissue cysts, the risks of oocyst exposure should not be downplayed. Outdoor cats should be prevented from accessing community gardens as a food biosecurity issue and exclude cats from any location where food is grown. Children should avoid areas where cat feces may be found, domestic cat access to the outdoors should be limited, and steps taken to reduce the number of free-roaming domestic cats and the associated number of *T. gondii* oocysts.

Widespread participation, especially with human and veterinary health practitioners, is necessary to stem the societal and ecosystem impacts of toxoplasmosis. Doctors, public health specialists, veterinarians, and even wildlife biologists should know to caution the public to always wash hands after working in any soil where cat feces may be found, exclude cats from any location where children or others play in a manner that might lead to hand-to-mouth contact with contaminated soil, and take steps to reduce the number of free-roaming domestic cats on the landscape. Children should be taught to wash their hands thoroughly after touching a pet that has access to the outdoors.

Local capacity also includes a commitment to laws to control the number of feral domestic cats on the landscape to minimize the risk of transmission of *T. gondii*. Current discussions surrounding animal sheltering, as discussed above, often dismiss this risk as minimal and almost never incorporate the clear society-level impacts from chronic infection that have now been shown in the literature for over a decade. Veterinary schools have a particular responsibility to educate their students on the risks of this disease and not to allow specialized programs with outside funding, e.g., “shelter medicine” programs funded by animal rights organizations, to put out messages that undermine established science. Such changes will be difficult, given that promoting unowned free-roaming domestic cats as perfectly acceptable features of the landscape has garnered significant funding. Progress to address this situation can come from a transdisciplinary, integrative approach that considers the substantial advances in research on *T. gondii* of recent years. In addition, controlling the feral cat population will have a positive conservation outcome for wildlife. For example, annual mortality of wild birds in the USA reaches 2.4 billion and 204 million in Canada due to feral cat predation, increasing the probability of population extinction or decline for some bird species. In addition, 6.3–22.3 billion mammals are killed each year in the USA (Loss et al. 2013, 2015). Comprehensive and sound policies and control interventions based on science are required to reduce these astronomical impacts.

## Conclusions

One Health has emphasized the need to bridge disciplines linking human health, animal health, and ecosystem health. Toxoplasmosis demands integrative approaches breaching disciplinary boundaries. This integration is needed to generate new approaches to manage and control the disease. The complexity of toxoplasmosis requires the development of a dashboard system of measures that are a combination of health and ecological indicators, that is, an easy set of indicators for quick reference to identify prevention and management needs.

Transdisciplinarity, integrative research, and capacity building are core elements in establishing One Health interventions that address toxoplasmosis. Innovative participatory methodologies that operationalize knowledge flow among stakeholders should consensually and sustainably address this major problem confronting society, wildlife, and ecosystems globally (Aguirre et al. 2019). The One Health approach to toxoplasmosis epidemiology and control requires practical, sustainable, and effective solutions with a keen understanding of local socioeconomic and cultural factors as well as a solid grasp of complex local, regional, national, and international health and environmental policies. One Health offers time-sensitive opportunities for practitioners to apply their expertise to give rise to simultaneous benefits for humans, animals, and the environment.
